# Associations between per- and polyfluoroalkyl substances (PFAS) and diabetes in two population-based cohort studies from Sweden

**DOI:** 10.1038/s41370-023-00529-x

**Published:** 2023-03-24

**Authors:** Linda Dunder, Samira Salihovic, Sölve Elmståhl, P. Monica Lind, Lars Lind

**Affiliations:** 1 Department of Medical Sciences, Occupational and Environmental Medicine, Uppsala University, Uppsala, Sweden; 2 School of Medical Sciences, Örebro University, Örebro, Sweden; 3 Division of Geriatric Medicine, Department of Clinical Sciences in Malmö, Lund University, Malmö, Sweden; 4 Department of Medical Sciences, Cardiovascular Epidemiology, Uppsala University, Uppsala, Sweden

**Keywords:** Perfluoroalkyl substances (PFAS), Diabetes, Cross-sectional, Longitudinal, Metabolomics

## Abstract

**Background:**

Per- and polyfluoroalkyl substances (PFAS) have been suggested to contribute to the development of metabolic diseases such as obesity, diabetes and non-alcoholic fatty liver disease (NAFLD). However, evidence from epidemiological studies remain divergent. The aim of the present study was to evaluate associations between PFAS exposure and prevalent diabetes in a cross-sectional analysis and fasting glucose in a longitudinal analysis.

**Methods:**

In 2373 subjects aged 45–75 years from the EpiHealth study, three PFAS; perfluorohexanesulfonic acid (PFHxS), perfluorooctanoic acid (PFOA) and perfluorooctane sulfonic acid (PFOS) were analyzed in plasma together with information on prevalent diabetes. Participants in the PIVUS study (*n* = 1016 at baseline, all aged 70 years) were followed over 10 years regarding changes in plasma levels of six PFAS; PFHxS, PFOA, PFOS, perfluorononanoic acid (PFNA), perfluorodecanoic acid (PFDA), and perfluoroundecanoic acid (PFUnDA), and changes in plasma levels of fasting glucose.

**Results:**

In the EpiHealth study, no overall associations could be observed between the levels of PFOA, PFOS or PFHxS and prevalent diabetes. However, there was a significant sex-interaction for PFOA (*p* = 0.02), and an inverse association could be seen between PFOA (on a SD-scale) and prevalent diabetes in women only (OR: 0.71, 95% CI: 0.52, 0.96, *p*-value: 0.02). This association showed a non-monotonic dose-response curve. In the PIVUS study, inverse relationships could be observed between the changes in levels (ln-transformed) of PFOA and PFUnDA vs the change in fasting glucose levels (ln-transformed) over 10 years (*p* = 0.04 and *p* = 0.02, respectively). As in EpiHealth, these inverse associations were significant only in women (PFOA: β: −0.03, *p* = 0.02, PFUnDA: β: −0.03, *p* = 0.03).

**Impact:**

Exposure to per- and polyfluoroalkyl substances (PFAS) has been linked to unfavorable human health, including metabolic disorders such as obesity, diabetes and non-alcoholic fatty liver disease. However, results from in vivo, in vitro and epidemiological studies are incoherent. The aim of the present study was therefore to investigate associations between PFAS and diabetes in a cross-sectional study and glucose levels in a longitudinal study. Results show inverse associations in women only. Results also display non-monotonic dose response curves (*i.e*., that only low levels of PFOA are related to higher probability of prevalent diabetes). This suggests that sex differences and complex molecular mechanisms may underlie the observed findings. A better understanding of the factors and molecular mechanisms contributing to such differences is recognized as an important direction for future research.

**Conclusions:**

PFOA was found to be inversely related to both prevalent diabetes and changes in plasma glucose levels among women only. Thus, our findings suggest there are sex differences in the inverse relationship of PFOA and type 2 diabetes and glucose levels.

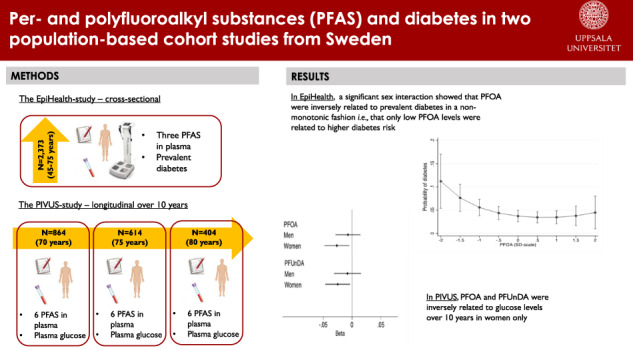

## Introduction

Per-and polyfluoroalkyl substances (PFAS) are a vast class of organic chemicals widely used in industrial and consumer products such as personal care products, cleaning solutions, cosmetics, textile and paper coatings, food packaging, firefighting foams and surfactants [1]. PFAS owe their properties to the extremely strong carbon-fluoride bond which makes them stable and persistent in the environment and in biological organisms. It has been demonstrated that PFAS can be detected in the blood of nearly all investigated individuals, including pregnant women and children in many populations studied worldwide [2–4]. Several PFAS have been put forward as emerging endocrine-disrupting chemicals (EDCs) due to their ability to act on a number of endocrine pathways. The most investigated PFAS are perfluorooctyl sulfonic acid (PFOS) and perfluorooctanoic acid (PFOA), with estimated serum half-lives of 5.4 and 3.8 years, respectively [5]. The main exposure sources for humans are food and drinking water (particularly in contaminated communities). In 2012 it was discovered that the drinking water in the city of Uppsala, Sweden was contaminated with PFAS, foremost PFOS and perfluorohexanesulfonic acid (PFHxS). Several studies from Sweden, and other countries, have shown that groups in the population that have been exposed to PFAS contaminated drinking water over a long period have elevated levels of PFAS in their blood, compared to groups that only have background exposure from other sources than drinking water [6–8].

PFAS exposures have repeatedly been shown to induce hyperglycemia and glucose metabolism related outcomes in various animal models. In mice, exposure to PFOA during 28 days produced insulin sensitivity and glucose tolerance [9], and developmental PFOS exposure has been shown to increase HOMA-IR index [10]. By combining data from in vivo studies (with both wild type and gene knockout mice) and in vitro studies (with both mouse islet β cells and human embryonic kidney cells) it has been reported that PFOS stimulates insulin secretion and intracellular calcium level by activating G protein-coupled receptor 40 (GPR40) both in mice and in human GPR40. The authors thus provided mechanistic insight on how PFAS exposure can disrupt insulin-secretion in humans [11].

In 2020, EFSA was commissioned by the European Commission to compile a scientific evaluation on the risks to human health related to the presence of PFAS in foods. In the report, EFSA concludes that there is insufficient evidence for associations between PFAS exposure and diabetes, obesity and non-alcoholic fatty liver disease (10.2903/j.efsa.2020.6223). However, in a scoping review from 2021, authors summarize 10 studies on PFAS exposure and type 1 and type 2 diabetes and found that 3 of these reported positive associations, 2 negative/inverse and 5 reported null findings. The authors of the review conclude: “*These data indicate the need for further studies to better assess these associations between PFAS and diabetes*” [12].

Thus, the primary aim of the present study was to investigate associations between plasma concentrations of PFHxS, PFOA and PFOS, and prevalent diabetes in a cohort of middle-aged women and men (The Epihealth study, Uppsala, Sweden). Additionally, in a supportive analysis of data from a cohort of elderly women and men (The PIVUS study, Uppsala, Sweden), we evaluated whether 10-year changes in plasma PFAS (PFOS, PFOA, PFHxS), perfluorononanoic acid (PFNA), perfluorodecanoic acid (PFDA) and perfluoroundecanoic acid (PFUnDA) levels were related to 10-year changes in plasma levels of glucose.

## Methods

### Ethical statement

The study was approved by the Ethics Committee of Uppsala University, and all the participants gave their informed consent prior to the study.

### Cohorts

Two population-based Swedish cohorts were utilized for the present study. The Epihealth study is a large cohort study including men and women in the age range 45–75 years from the Swedish general population, as described in detail previously [13]. Briefly, participants were randomly selected from the population registries of the Swedish cities Malmö and Uppsala between 2011 and 2016 with the response rate of approximately 20%. The present study includes a subset of 2373 individuals for whom data exists on both PFAS measurements (metabolomics) and prevalent diabetes between the years 2011 and 2016.

The Prospective Investigation of the Vasculature in Uppsala Seniors (PIVUS) study is a longitudinal investigation over 10 years. A total of 1016 subjects (50% women), aged 70 years and living in Uppsala, were investigated at baseline during 2001–2004. The participants were invited to follow-up examinations at age 75, (*n* = 822), and at 80 years (*n* = 603). During the first 5 years of the study, 52 individuals passed away and 142 withdrew. During the next 5 years, 106 individuals passed away and 113 subjects withdrew. All measurements were carried out with essentially the same protocol at age 70, 75 and 80 years. More detailed information on the study population can be found in Lind et al. 2005 [14].

### Physical examinations

Across both cohorts, body mass index (BMI) was calculated as weight in kilograms divided by the square of body height in meters (kg/m^2^). In EpiHealth, weight was measured on a scale that uses bioelectrical impedance analysis to also calculate total fat mass. Total fat mass was then divided by total body weight (Tanita body composition analyzer BC-418MA, Tokyo, Japan).

### Questionnaires

In EpiHealth, a web-based questionnaire about medical and family history and symptoms as well as lifestyle factors, including diet (*e.g*., fish intake), was filled in by all participants. Fish intake was converted to grams per day using the consumption frequencies of different kinds of fish (cod, tuna, salmon, etc.) using standardized portion sizes. Participants also reported medication usage, leisure time, and physical activity in five levels from low (level 1) to strenuous physical activity (level 5). They also reported age, sex, alcohol intake (drinks per week), education length (up to 9 years, 10–12 years, or >12 years), and current smoking habit (smoked years in life).

In PIVUS, the participants were asked to answer a questionnaire regarding their socioeconomic status, medical history, physical activity, smoking habits and regular medication. Unfortunately, no data on dietary intake was collected in the PIVUS study.

### Healthy lifestyle index (HLI) in EpiHealth

We have previously constructed a healthy lifestyle index (HLI) to use as a confounder in our analyses. The HLI includes physical activity (levels 4 and 5 considered healthy), healthy diet (upper 25% of individuals in adherence to a previously described healthy dietary pattern), sleeping habits (7–9 h of sleep considered healthy), alcohol intake (>1 drink/day considered unhealthy), stress (never or rarely stressed considered healthy), smoking (smoked <2 years in life defined as non-smoker).

### PFAS analyses

In Epihealth, 100 mL of blood was taken, and 90 mL was prepared into plasma, serum and whole blood (for later DNA extraction) and stored in −80 °C in a biobank facility for later PFAS analysis. Blood samples from 2373 individuals were randomly chosen for PFAS analysis and PFAS levels could be detected in >95% of the study population. PFAS (PFHxS, PFOA and PFOS) were analyzed in plasma by non-targeted metabolomics (Metabolon Inc, Morrisville, NC; UAS). After the analysis, the PFAS values were normalized and expressed in relative concentrations. In a third cohort (the POEM study, Uppsala, Sweden, 502 males and females, all aged 50 years) PFAS were measured both with the same relative technique as used in EpiHealth and with the same quantitative technique using standards as used in the PIVUS study. When linear regression analysis were performed, the fit between the relative and quantitative technique was very good with correlations coefficients ranging from 0.83–0.96 for PFOS, PFOA and PFHxS. From those linear regression models, formulas could be derived to estimate quantitative values for the values being initially measured on the relative scale. The same formulas were then used in the EpiHealth study to give a rough estimate of the median levels of PFOS, PFOA and PFHxS in that cohort.

In the PIVUS study, blood serum and plasma were collected in the morning (8–10 am) after an overnight fast and stored in freezers (−70 °C) until later analysis. The current study evaluated six PFAS for which >75% of the study population showed measurable levels above the lower limit of detection (LOD); PFHxS, PFOA, linear isomer of PFOS, PFNA, PFDA, and PFUnDA. PFAS levels were analyzed by UPLC-MS/MS as previously described [15]. The method detection limits (MDLs) for all three investigations ranged from 0.01–0.18 ng mL^−1^ depending on the analyte. PFAS values below LOD were replaced by LOD/√2.

### Outcome assessment

In EpiHealth, prevalent diabetes was defined as either taking diabetes medication or having fasting glucose levels of ≥7 mmol/L. In PIVUS, we could not use incident diabetes as outcome due to the limited number of cases. Plasma glucose levels were analyzed by routine laboratory methods at Uppsala University Hospital, Uppsala, Sweden after an overnight fast.

### Statistical analyses

STATA 16 was employed for all computations (Stata Corp, College Station, TX, USA).

#### Analyses of EpiHealth data

The three PFAS were inverse-ranked normalized to ensure a normal distribution and to obtain all the PFAS on the same SD-scale. Thus, the ORs reported for diabetes are for a 1 SD change of each measured PFAS. For the EpiHealth data, logistic regression analysis was used to evaluate the relationships between the relative concentrations of PFHxS, PFOA and PFOS, and prevalent diabetes. The fully adjusted model was adjusted for age, sex, participation date, total fat mass, smoking, education, physical exercise, alcohol use, creatinine as a proxy for kidney function and fish intake. An additional adjustment was also made for HLI and for plasma levels of the marine omega-3 fatty acids eicosapentaenoic acid (EPA) and docosahexaenoic acid (DHA).

A sex*PFAS interaction term was inserted in the main models in order to investigate potential sex differences. If the interaction term was significant, the analyses were also performed in men and women separately. Further, age-interactions were investigated by inserting an age*PFAS interaction term in the main models. Finally, potential non-monotonic relationships were examined by inserting a squared term of the PFAS in the models. We divided the nominal *p*-value by 3 to account for 3 different measured PFAS (0.05/3 = 0.016666), which resulted in a significance level of *p* < 0.016666 for the associations between PFAS and prevalent diabetes in the primary analysis. This Bonferronni-adjustment for multiple testing was done to limit the number of false positive findings frequently reported in the literature.

#### Analyses of PIVUS data

Due to the right-skewed distribution, plasma levels of all PFAS and plasma glucose levels were ln-transformed in order to obtain a more normal distribution of the data. First we wanted to see if there was a significant change in glucose levels over the 10-year follow-up period. Thus, the change in plasma glucose levels over 10 years (three measurements) was assessed by mixed-effects linear regression models with fasting glucose as the dependent variable, time as the independent variable, and sex as confounder (age was the same in all subjects). The same analysis has been performed also for the changes in PFAS levels over 10 years and the results have been previously described [16].

Thereafter, we examined associations between the changes over 10 years in plasma levels of both total PFAS and individual PFAS: PFHxS, PFOA, linear isomer of PFOS, PFNA, PFDA, and PFUnDA (three measurements) and the changes over 10 years in fasting glucose levels (three measurements). The theory and assumptions behind this model as well as the detailed formula can be found in [17]. The general formula is; Y_ij_ = Z_i_beta_0_ − X_i1_beta_C_ + (X_ij_ − X_i1_)beta_L_ + e_ij_, where Y is fasting glucose levels, X is the specific PFAS, i is the individual, j the time, beta_C_ is the coefficient for the first observation and beta_L_, is the coefficient for the change over time. Confounders and the intercept are given as Z_i_beta_0_. The general formula used in STATA for the calculations of the present study was; mixed fasting glucose PFASchange PFAS1 sex | |id:, where 1 denotes the first observation at age 70.

The fully adjusted model was adjusted for sex, BMI, insulin and diabetes medication, smoking, physical exercise and glomerular filtration rate, (age was the same in all individuals). Additional adjustment was made for relative plasma levels of the marine omega-3 fatty acids EPA and DHA. The PIVUS study was regarded as a supportive analysis and *p* < 0.05 was thus regarded as statistically significant.

## Results

### The EpiHealth study – cross-sectional analyses

The study population included a random subset of 2373 individuals from the EpiHealth study with investigations on both PFAS levels and prevalent diabetes between the years 2011 and 2016. General and clinical characteristics for the study population are given in Table 1. Participant age was 61.1 ± 8.4 and 50.6% were men. Prevalent diabetes was 8.4% for the whole study population, and there was a significant difference between men and women (12 and 4.7 %, respectively, *p* < 0.0001). The estimated relative plasma PFAS concentrations (ng/mL) were the following: PFOS: 8.2; PFOA: 2.2; PFHxS: 5.5.

**Table 1:** General and clinical characteristics reported as percent or mean ± standard deviation (SD) for the EpiHealth study population, *n* = 2373.

Characteristic	Category	Mean ± SD
Sex (%)	Men	50.6
	Women	49.4
Age		61.1 ± 8.4
Smoking status	Years in life	6.7 ± 8.9
Leisure time physical activity (%)	Sedentary	3.3
	Mostly sedentary	24.3
	30 min walk/day	39.5
	Strenuous activity 1–2 h a week	26.2
	Strenuous activity 60 min/day	6.7
Education (%)	Primary and elementary school	21.4
	Secondary school	28.5
	University	50.1
Alcohol	Drinks/day	2.4 ± 2.9
Fish intake	Grams/day	31.8 ± 19.2
BMI, (kg/m^2^)		26.5 ± 3.8
Total fat mass (%)		30.2 ± 8.1
Prevalent diabetes (%)		8.4
Men (%)		12
Women (%)		4.7

**Fig. 1:**
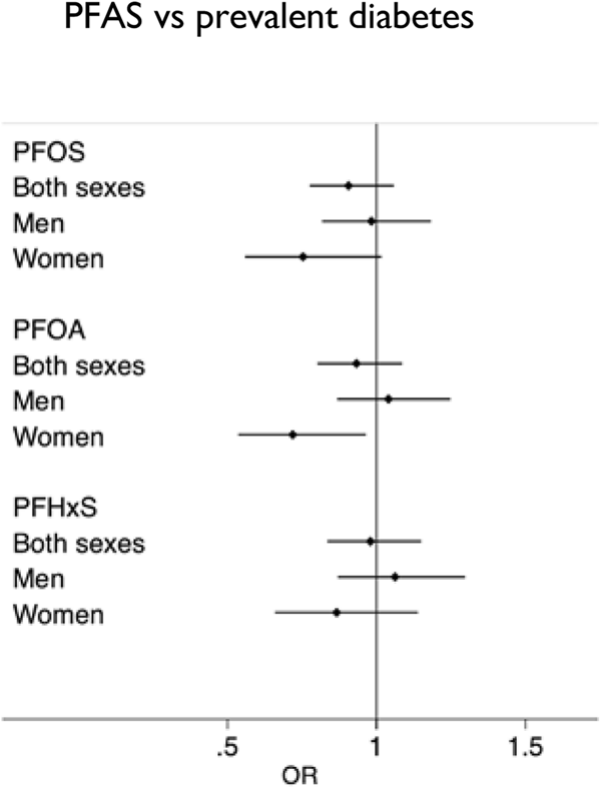
Associations between PFAS exposure and prevalent diabetes in the Epihealth study. Forest plot of the associations between perfluorooctane sulfonic acid (PFOS), perfluorooctanoic acid (PFOA), perfluorohexanesulfonic acid (PFHxS) (all on a standard deviation (SD) scale) and prevalent diabetes. Black dots represent odds ratios and lines represent 95% confidence intervals (CI). OR; odds ratio. The ORs reported for diabetes are for a 1 SD change of each measured PFAS.

**Fig. 2:**
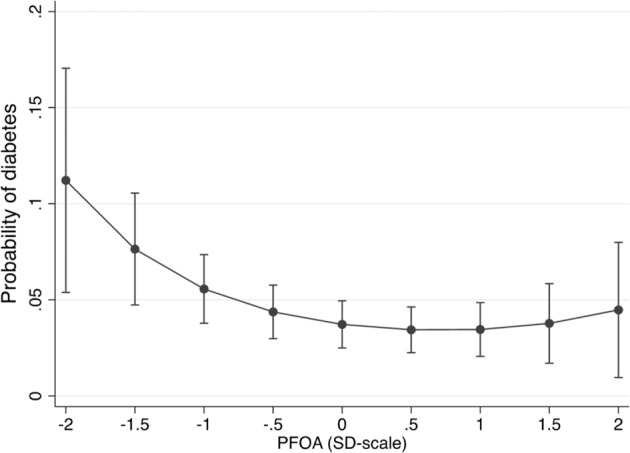
The probability for prevalent diabetes for given values of perfluorooctanoic acid (PFOA) in the EpiHealth study. The probability (and 95% confidence interval) for prevalent diabetes was calculated for given values for PFOA on a standard deviation (SD) scale (*n * = 2373). The figure shows that the association displays a non-monotonic dose-response relationship.

In Epihealth, no overall significant associations could be observed between the levels of PFOA, PFOS or PFHxS and prevalent diabetes after full adjustment for sex, age, participation date, total fat mass, smoking, education, physical exercise, alcohol use and fish intake (Fig. 1 and Table S1). Additional adjustment for HLI did not alter the estimates substantially. The analysis did not reveal any age interactions for PFAS (data not shown). However, a significant sex interaction was observed for PFOA (*p* = 0.02), and an inverse association could be seen between PFOA levels (on a SD-scale) and prevalent diabetes in women only (Men: OR: 1.03, 95% CI: 0.84, 1.24, *p*-value: 0.80) (Women: OR: 0.71, 95% CI: 0.52, 0.96, *p*-value: 0.02). This association displayed a non-monotonic dose response curve, *i.e*., that the lowest PFOA levels were related to highest probability of prevalent diabetes (Fig. 2). This relationship was not altered if a measurement of the concentration of omega-3 fatty acids in plasma was included in the model (mean value for EPA plus DHA was 0.50 mmol/l (SD 0.15)) (measured by nuclear magnetic resonance spectroscopy (NMR) performed by Nightingale Health, Helsinki, Finland). Further adjustment for fruit intake (mean 118 (SD 80) g/day) and intake of vegetables (mean 113 (SD 62) g/day) did not have any impact on the estimate.

When we performed the analysis described above separately for the two outcomes diabetes medication (*n* = 60) and increased fasting glucose without medication (*n* = 139), the estimates for PFOA in women were almost identical for the two outcomes, although the confidence interval was larger for the outcome diabetes medication, possibly due to the smaller number of cases for that outcome (OR: 0.69, 95% CI: 0.37, 1.25 for the diabetes medication outcome and OR: 0.70, 95% CI: 0.50, 0.96 for the increased fasting glucose without medication outcome).

### The PIVUS study – longitudinal analyses

The study population included 1016 elderly individuals (50.1% women) within the PIVUS cohort that were investigated three times over a 10-year follow-up. General and clinical characteristics of the population at baseline are provided in Table 2. Time trends for the PFAS levels have been previously described in detail [16]. In short, over the 10-year follow-up period there was an increase in concentrations of PFHxS, PFUnDA, PFNA, and PFDA (7% to 34%), whereas decreased concentrations could be seen for PFOSA, PFHpA, PFOS, and PFOA (−75% to −27%). Levels of the different PFAS were in the following ranges: PFHxS: 2.05–3.13, PFUnDA: 0.28–0.44, PFNA: 0.70–1.04, PFDA: 0.31–0.42, PFHpA: 0.03–0.06, PFOS: 7.24–13.26, PFOA: 2.51–3.79 ng/mL [16]. For fasting glucose levels there were no significant changes over the 10-year follow-up period (β: −0.0009, *p* = 0.40), and there was no significant difference regarding this between men and women (*p* = 0.33 for the sex-interaction term).

**Table 2:** Baseline characteristics of the PIVUS study population at age 70 (*n* = 1,016), 75 (*n* = 822) and 80 (*n* = 603) years, reported as percent (%), mean ± standard deviation (SD).

		70 years (*n* = 1,016)	75 years (*n* = 822)	80 years (*n* = 603)
Characteristic	Category	Mean ± SD	Mean ± SD	Mean ± SD
Current smoking	(%)	11	6	3
Physical activity	Sedentary	12	12	13
	Light	59	62	67
	Moderate	22	21	16
	Athlete	7	5	4
BMI, (kg/m^2^)		27.0 ± 4.3	26.9 ± 4.4	26.9 ± 4.5
Fasting glucose, (mmol/L)		5.34 ± 1.61	5.24 ± 1.47	5.28 ± 1.38
Insulin medication, (%)		2	3	3
Diabetes medication, (%)		6	9	9
GFR, (mL/min/BSA)		86.3 ± 16.4	70.8 ± 13.7	62.2 ± 14.7

*BMI* body mass index, *GFR* glomerular filtration rate.

When concentrations of all the six PFAS were summed up and ln-transformed before analysis, the change in this summary measurement of PFAS was not significantly related to the change in glucose (β: −0.012, 95% CI: −0.027, 0.0032, *p* = 0.12) after full adjustment.

**Fig. 3:**
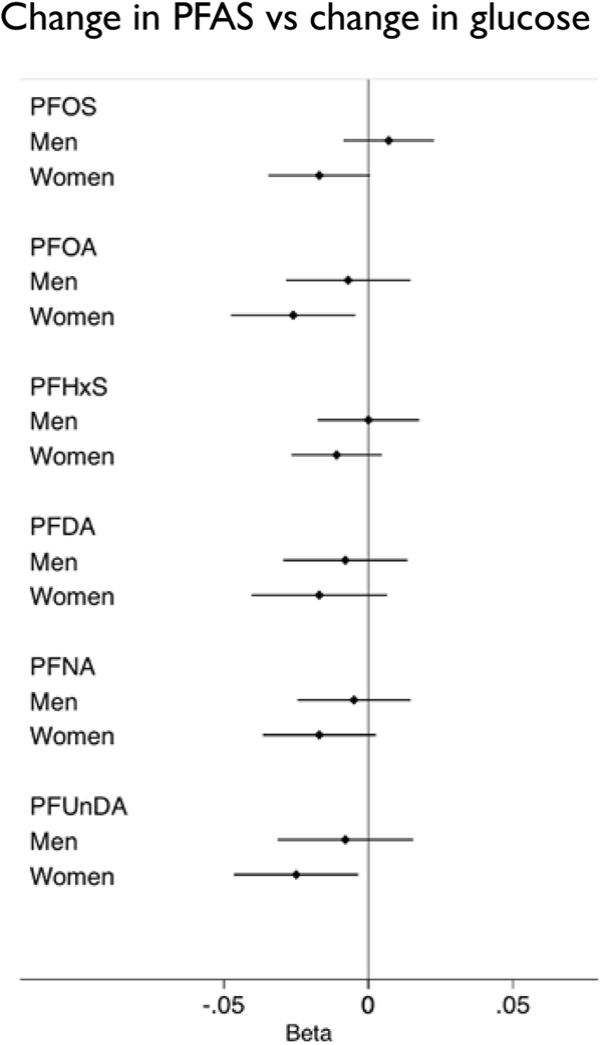
Associations, over 10 years, between changes in PFAS exposure and changes in glucose levels in the PIVUS study. Forest plot of the associations between changes in plasma levels of perfluorooctane sulfonic acid (PFOS), perfluorooctanoic acid (PFOA), perfluorohexanesulfonic acid (PFHxS), perfluorodecanoic acid (PFDA), perfluorononanoic acid (PFNA), perfluoroundecanoic acid (PFUnDA) (all ln-transformed), and changes in plasma levels of fasting glucose (ln-transformed) over a 10-year follow-up period. Black dots represent beta values and lines represent 95% confidence intervals. Beta; regression coefficient.

The six PFAS were then investigated one by one. After full adjustment, inverse relationships could be observed between the changes in levels of PFOA and PFUnDA (ln-transformed), and the change in plasma glucose levels (ln-transformed) over 10 years (*p* = 0.04 and *p* = 0.02). Since beta-values on a ln-scale are hard to interpret, we performed calculations to give an example where a 1 ng/ml increase in PFOA (or PFUnDA) corresponds to a 0.037 mmol/l reduction in fasting glucose (calculated from beta values in Table 3). A summary of all the associations is also presented as a forest plot (Fig. 3). In addition, when a term for interaction between sex and the change in PFAS levels was inserted in the models, significant interactions with the change in fasting glucose levels were seen for the changes in PFOA and PFUnDA, and the inverse associations could be observed in women only (PFOA: β: −0.03, *p* = 0.02, PFUnDA: β: −0.03, *p* = 0.03) (Table 3). The inverse relationship seen between the change in glucose and changes in PFOA and PFUnDA in women were not altered by the inclusion of the proportion (relative percentage of the total amount of fatty acids analyzed, *i.e*., a relative measure) of omega-3 fatty acids in plasma in the model (mean value for EPA plus DHA was 3.3% (SD 1.2)) (measured by gas chromatography) as described in [18].

**Table 3:** Associations between changes in plasma levels of PFAS and changes in fasting glucose levels during 10 years of follow-up.

	Adjusted			Adjusted also for BMI			Sex interaction			Women only			Men only		
	β	95% CI	*p*-value	β	95% CI	*p*-value	β	95% CI	*p*-value	β	95% CI	*p*-value	β	95% CI	*p*-value
**PFOS**	−0.003	−0.014, 0.009	0.60	−0.003	−0.01, 0.009	0.62	−0.004	−0.01, 0.004	0.28	−0.02	−0.04, −0.002	0.05	0.007	−0.009, 0.02	0.39
**PFOA**	**−0.02**	**−0.04, −0.004**	**0.04**	**−0.02**	**−0.04, −0.004**	**0.046**	**−0.01**	**−0.02, −0.002**	**0.02**	**−0.03**	**−0.05, −0.01**	**0.02**	−0.007	−0.03, 0.01	0.49
**PFHxS**	−0.008	−0.02, 0.004	0.22	−0.006	−0.02, 0.006	0.34	−0.006	−0.01, 0.002	0.12	−0.01	−0.03, 0.006	0.17	0.001	−0.02, 0.02	0.97
**PFNA**	−0.009	−0.02, 0.007	0.25	−0.009	−0.02, 0.005	0.23	−0.008	−0.02, 0.002	0.11	−0.02	−0.04, −0.0004	0.12	−0.005	−0.02, 0.01	0.66
**PFDA**	−0.01	−0.03, 0.006	0.12	−0.01	−0.03, 0.006	0.17	−0.008	−0.02, 0.002	0.11	−0.02	−0.04, −0.0004	0.14	−0.008	−0.03, 0.01	0.48
**PFUnDA**	**−0.02**	**−0.04, −0.004**	**0.02**	**−0.02**	**−0.04, 0.004**	**0.047**	**−0.01**	**−0.02, −0.0002**	**0.02**	**−0.03**	**−0.05, −0.01**	**0.03**	−0.008	−0.03, 0.02	0.52

The fully adjusted model was adjusted for sex, participation time, BMI, insulin and diabetes medication, smoking, physical exercise and glomerular filtration rate (age was the same in all individuals). Significant *p-* values and associated results are marked in bold.

*β* beta (regression coefficient), *PFHxS* perfluorohexane sulfonic acid, *PFOS* perfluorooctanesulfonic acid, *PFOA* perfluorooctanoic acid, *PFNA* perfluorononanoic acid, *PFDA* perfluorodecanoic acid, *PFUnDA* perfluoroundecanoic acid, *SE* standard error.

In an additional sensitivity analyses where all observations of participants on antidiabetic medication were excluded from the analysis, the change in fasting glucose was still inversely related to the changes in PFOA and PFUnDA in women (β: −0.02, *p* = 0.047 for PFOA and β: −0.018, *p* = 0.044).

## Discussion

### Main findings

The cross-sectional analyses conducted in middle-aged men and women in the EpiHealth study did not reveal any overall significant associations between the three investigated PFAS; PFHxS, PFOA and PFOS, and prevalent diabetes. However, there was a significant sex interaction for PFOA, and an inverse association could be seen between PFOA levels and prevalent diabetes in women only. This association displayed a non-monotonic dose response curve, *i.e*., the lowest PFOA levels were related to the highest probability of prevalent diabetes but no elevated risk of diabetes could be seen with increasing PFAS exposure.

Similarly, in the longitudinal analyses of data from the PIVUS study, changes in levels of PFOA and PFUnDA were inversely associated with the change in fasting glucose levels over 10 years, also in women only.

Taken together, a sex difference regarding PFAS exposure and diabetes outcomes could be observed in both cross-sectional and longitudinal analyses.

### Comparison with previous studies

Overall, the scientific evidence of links between PFAS exposure and risk of developing diabetes is contradictory. This is clear from a review by Margolis et al. from 2021 showing that many studies investigating the relationship between PFAS exposure and diabetes report positive associations, but there are also reports of negative and inverse associations [12]. To date, the majority of studies with human evidence are cross-sectional in nature. For example, a large cross-sectional study from the C8 Health project (*n* = 60,439; 4291 T2D cases) found that PFAS levels were significantly lower in individuals with T2D than in those without T2D and lowest in those with type 1 diabetes [19]. Similarly, a smaller case-control study (*n* = 13,922; 1055 cases of T2D), also from the C8 Health Project including long-time residents in an area with PFAS contaminated drinking water, showed a decreased risk of prevalent T2D in individuals with high PFOA concentrations [20]. Concomitantly, Su et al. observed that high exposure to PFOA, PFNA and PFUA was related to decreased risk of prevalent T2D and improved glucose homeostasis, while the opposite could be seen for PFOS exposure [21]. In line with the positive association seen for PFOS in the aforementioned study, our research group have also previously reported positive associations between PFNA exposure and prevalent diabetes in elderly individuals from the PIVUS study [22].

The concentration of PFAS in the blood may be altered by factors related to diabetes diagnosis such as changes in diet, and other treatment factors can influence exposure, distribution or excretion of PFAS. Thus, there is always a potential problem of reverse causation in cross-sectional studies, which stresses the importance of supportive data from prospective studies where all individuals are free from T2D at baseline.

However, the limited number of prospective studies published so far have also arrived at conflicting findings. A nested case-control study with 793 incident T2D cases from the Nurses’ Health Study II in USA revealed that higher plasma concentrations of PFOS and PFOA (the two PFAS found in highest concentrations) were linked to an elevated risk of developing T2D with ORs of 1.62 (95% CI: 1.09, 2.41; *p*_trend_ = 0.02) and 1.54 (95% CI: 1.04, 2.28; *p*_trend_ = 0.03), respectively [23]. In contrast, another prospective nested control study from Sweden reported an odds ratio of 0.52 (95% confidence interval, CI: 0.20, 1.36) of T2D for the sum of PFAS exposure. In addition, higher PFAS exposure was also associated with lower insulin resistance [24]. This is in line with the results of the present study, which exhibit a prospective inverse association between PFOA and PFUnDA exposure and glucose levels among women. When it comes to studies investigating links between PFAS exposure and glucose homeostasis parameters, these results are also contradictory with reports of no evidence of such links [22, 25, 26], but also positive [27, 28], and inverse associations [29, 30]. As far as we know, there are only two previous studies with a prospective design [31, 32]. In one of these studies, plasma levels of several PFAS were positively associated with markers of insulin resistance and β-cell function at baseline in adults with high risk of developing T2D. However, this could not be seen during the prospective 4.6-year follow-up [31]. Within the Study of Women’s Health Across the Nation Multi-Pollutant study (SWAN-MPS) 1237 women free of diabetes were followed from 1999–2000 to 2017 and then examined in terms of PFAS concentrations and incident diabetes. The results showed that higher levels of PFOA, PFHxS, PFOS and 2-(*N*-methyl-perfluorooctane sulfonamido) acetic acid (MeFOSAA) were related to a higher risk of incident diabetes. In addition, combined exposure to seven PFAS was related to an even higher hazard ratio (HR) of 2.62 (95% CI 1.12, 6.20), than for each PFAS alone (HR range 1.36–1.85), which indicates an additive or synergistic effect of multiple PFAS exposure on diabetes risk [32].

### Non-monotonic dose response curves (NMDRCs)

Non-monotonic dose response curves (NMDRCs), often displayed as a U- or inverted U-shape, are common for natural hormones, vitamins and pharmacological compounds. In addition, as the study of low-dose effects of EDCs has developed during the last two decades, studies have repeatedly identified NMDRCs in EDC-exposed cultured cells, laboratory animals and human populations [33, 34]. In the present study we observed a non-monotonic relationship between PFOA levels and prevalent diabetes in the EpiHealth study, with an inverse dose response at low doses, while turning into a direct association at higher levels. It seems that only low levels of PFOA are related to a higher probability of prevalent diabetes in the present study (Fig. 2). Concomitantly, our research group has previously found a NMDRC between PFNA and prevalent diabetes also in the PIVUS study [22]. This is in line with a large prospective cohort study of around 71,000 women which report an inverse U-shaped association between estimated dietary PFOA exposure and the risk of developing T2D over more than 15 years of follow-up [35]. In a review on potential health risks of PFOA exposure it is summarized that the dose-response curve between PFOA and several outcomes presents a steeper slope at low levels than at higher levels, which corresponds to the results of the present study and once again points out the importance of investigating low-dose effects of EDCs [36].

### Sex-specific effects

One of the reasons for the divergent findings regarding associations between PFAS exposure and diabetes in different cohort studies could be that few have considered sex differences. In addition, many of the previous studies investigating the link between PFAS and diabetes have focused on gestational diabetes, thus generating a natural sex differentiation, focusing only on women’s health. In the present study we included a sex-interaction term in the models in order to detect any potential sex-specific effects regarding the associations between PFAS levels and prevalent diabetes and glucose levels. In EpiHealth, the association between PFOA levels and prevalent diabetes was significant in women only. Concomitantly, the relationships between the change in PFOA and PFUnDA levels and the change in glucose levels over 10 years were found in women only from the PIVUS study. We have previously reported sex-specific effects regarding PFAS levels and measures of body composition in these two cohorts and in another cohort from the same town, the Prospective study on Obesity, Energy, and Metabolism (POEM) study [37]. Sex-specific effects have also been reported when it comes to links between PFAS and other outcomes such as deteriorated vaccine response [38] and markers of carotid artery atherosclerosis [39]. The potential mechanisms underlying the observed sex interactions are largely unknown but could arise from sex-specific differences in genotype, body size and hormonal status (menstruation, pregnancy, lactation, and menopause) [40–42], but also differences in chain length or mode of action among PFAS themselves [43]. The clinical implications of the inverse association between PFOA and PFUnDA with type 2 diabetes and blood glucose levels among women are unknown. The findings indicate a difference between men and women that may be relevant to sex ratios of disease. New studies are necessary to define mechanisms and implications of the observed sex differences.

### Diet as an important confounding factor

Dietary intake has been shown to be the main route of PFAS exposure in humans, and diet is also an important factor in diabetes etiology, and of course linked with lifestyle factors. Thus, potential confounding by diet or lifestyle factors associated with diet must be taken into consideration when assessing the relationship between PFAS exposure and diabetes risk [44]. Throughout the world there are considerable differences in which PFAS are detected and their concentrations in different food items. Thus, the dietary exposure differs across populations [45], and this together with differences in dietary habits could be one explanation for the discordant results reported in the existing epidemiological studies. EFSA has identified that healthy food groups including fish/shellfish, fruit and fruit products, and egg and egg products contributes most to PFOA, PFNA, PFHxS and PFOS exposure in the general population [46]. Concomitantly, a recent study on Swedish adolescents found that individuals adhering to a healthy and diverse diet had on average higher exposure levels of PFNA, PFDA, PFUnDA and PFOS than those eating less healthy foods. Authors conclude that these associations were most likely driven by a positive relation between seafood consumption and the healthy and diverse diet scores [47]. In line with this, individuals in an elderly Swedish population with high levels of PFAS were the ones with high adherence to a Mediterranean-like diet characterized by relatively high fish consumption [48]. In the present study we adjusted for confounding by fish intake in the analysis of Epihealth data in the full model, but also made additional adjustment for HLI (which includes a healthy diet score) and for plasma levels of the marine omega-3 fatty acids EPA and DHA in a separate model, but these additional adjustments did not affect the estimates. Nonetheless, we can never rule out the chance of residual or unmeasured confounding by a healthy diet. Unfortunately, there are no available data on diet in the PIVUS study, and we could therefore not make any adjustments for a healthy diet score in those analyses. However, we did have access to fatty acid measurements also in the PIVUS study and were therefore able to adjust for the proportion of plasma EPA plus DHA in our analyses, but this adjustment did not alter the results.

### Strengths and limitations

The strengths of the present study include a quite large sample from the EpiHealth study in which we have measurements on three different PFAS and prevalent diabetes. This together with supportive data from a longitudinal study of the change in levels of six PFAS (three measurements) and change in fasting glucose levels (three measurements) over 10 years in the PIVUS study. Results from both cohorts point in the same direction with inverse associations between some PFAS and prevalent diabetes and plasma glucose levels.

One limitation of the study is that we do not have incident cases of diabetes in the EpiHealth study. However, we do cover the longitudinal aspect of glucose metabolism in the PIVUS study. Another limitation is that we only have a relative quantification of PFAS levels in EpiHealth, while we have absolute PFAS concentrations in PIVUS. A relative quantification does not influence the associations, but it does limit our ability to directly compare the level of PFAS exposures in EpiHealth with PIVUS and other studies. Further, considering the importance of confounding by diet (especially sea food) when it comes to PFAS exposure, the lack of diet data in the PIVUS study (aside from plasma EPA plus DHA) is an important limitation to mention. Another potential limitation is also the well-known risk of selection bias linked to voluntary participation such as in the present study. It has been shown in previous epidemiological studies that this can lead to a selection of healthy individuals with a high socioeconomic status. Whether or not this would influence the results is however unknown. Since there is a possibility that diabetes treatment could affect the relationship between PFOA and diabetes diagnosis in women, we evaluated this by using two separate outcomes; diabetes medication and increased fasting glucose without medication. However, since the estimates were almost identical for these two outcomes, it is highly unlikely that diabetes medication as such would induce the relationship between PFOA and diabetes diagnosis in women. This assumption is further strengthened by the findings in the PIVUS study, when observations on antidiabetic treatment were excluded from the analysis. Lastly, the cohorts include middle-aged and elderly men and women from Sweden, which limits the generalizability to other age and ethnic groups.

In conclusion, concentrations of PFOA were inversely related to prevalent diabetes among women only in the EpiHealth study. Concomitantly, we also found that higher plasma concentrations of PFOA and PFUnDA were associated with lower levels of fasting glucose over a 10-year follow-up period among women in the PIVUS study. The fact that inverse associations in both the cross-sectional and longitudinal setting were observed among women only, and that the relationship between PFOA levels and prevalent diabetes displayed a non-monotonic dose response curve (i.e., that low levels of PFOA are related to highest probability of prevalent diabetes) suggests that sex differences and complex molecular mechanisms may underlie the observed findings. A better understanding of the factors and molecular mechanisms contributing to such differences is recognized as an important direction for future research.

### Supplementary information

Supplementary information

Reporting Checklist

## Data Availability

The datasets generated during and/or analyzed during the current study are available from the corresponding author on reasonable request.
