# Supplementary material for: Associations between per- and polyfluoroalkyl substances (PFAS) and diabetes in two population-based cohort studies from Sweden
Source: J Expo Sci Environ Epidemiol. 2023 Mar 24;33(5):748–56. doi: 10.1038/s41370-023-00529-x (PMC10541316; doi:10.1038/s41370-023-00529-x)
Supplement: Supplementary file 1 — Supplementary information [file 41370_2023_529_MOESM1_ESM.docx]

**Associations between per- and polyfluoroalkyl substances (PFAS) and diabetes in two population-based cohort studies from Sweden**

Linda Dunder^1^; Samira Salihovic^2^; Sölve Elmståhl^3^; P. Monica Lind^1^; Lars Lind^4^

**Affiliations:**

^1^ Department of Medical Sciences, Occupational and Environmental Medicine, Uppsala

University, Uppsala, Sweden

^2^ School of Medical Sciences, Örebro University, Örebro, Sweden

^3^ Division of Geriatric Medicine, Department of Clinical Sciences in Malmö, Lund University, Malmö, Sweden

^4^ Department of Medical Sciences, Cardiovascular Epidemiology, Uppsala University,

Uppsala, Sweden

**Table of contents**

**Tables**

**Table S1**. Associations between plasma levels of perfluorohexanesulfonic acid (PFHxS), perfluorooctanoic acid (PFOA) and perfluorooctane sulfonic acid (PFOS) and prevalent diabetes. The fully adjusted model was adjusted for sex, age, participation date, total fat mass, smoking, education, physical exercise, alcohol use and fish intake.

**Table S1**. Associations between plasma levels of perfluorohexanesulfonic acid (PFHxS), perfluorooctanoic acid (PFOA) and perfluorooctane sulfonic acid (PFOS) and prevalent diabetes. The fully adjusted model was adjusted for sex, age, participation date, total fat mass, smoking, education, physical exercise, alcohol use and fish intake.

|  |  |  |  | **Prevalent diabetes** | | |  |  |  |  |
| --- | --- | --- | --- | --- | --- | --- | --- | --- | --- | --- |
|  | OR-crude | 95 % CI | *p*-value | OR-adjusted | 95 % CI | *p*-value | β sex interaction | *p*-value sex interaction | β squared term | *p*-value squared term |
| **PFHxS** | 0.98 | 0.83, 1.15 | 0.80 | 1.04 | 0.88, 1.23 | 0.65 | -0.23 | 0.17 | 0.02 | 0.76 |
| **PFOA** | 0.93 | 0.80, 1.08 | 0.37 | 0.93 | 0.79, 1.09 | 0.37 | **-0.40** | **0.02** | **0.10** | **0.03** |
| **PFOS** | 0.91 | 0.76, 1.06 | 0.21 | 0.96 | 0.82, 1.13 | 0.61 | -0.30 | 0.06 | 0.09 | 0.06 |

Significant *p*-values and associated results are marked in bold. Note: β; beta (regression coefficient), CI; confidence interval, OR; odds ratio.
